# Performance of Five Food Regimes on *Anopheles gambiae* Senso Stricto Larval Rearing to Adult Emergence in Insectary

**DOI:** 10.1371/journal.pone.0110671

**Published:** 2014-10-23

**Authors:** Happiness S. Kivuyo, Paschal H. Mbazi, Denis S. Kisika, Stephen Munga, Susan F. Rumisha, Felister M. Urasa, Eliningaya J. Kweka

**Affiliations:** 1 Department of Zoology and Wildlife Conservation, College of Natural and Applied Sciences, University of Dar-es-salaam, Dar-es-salaam, Tanzania; 2 Centre for Global Health Research, Kenya Medical Research Institute, Kisumu, Kenya; 3 National Institute for Medical Research, Dar es Salaam, Tanzania; 4 Tropical Pesticides Research Institute, Division Of Livestock And Human Diseases Vector Control, Mosquito Section, Arusha, Tanzania; 5 Department of Medical Parasitology and Entomology, Catholic University of Health and Allied Sciences, Mwanza, Tanzania; Rosalind Franklin University, United States of America

## Abstract

**Background:**

Rearing of *Anopheles gambiae* s.s mosquitoes in insectary with quality cheap food sources is of paramount importance for better and healthy colony. This study evaluated larval survival and the development rate of aquatic stages of *An.gambiae* s.s under five food regimes; tetramin fish food (a standard insectary larval food), maize pollen, Cerelac, green filamentous algae and dry powdered filamentous algae.

**Methods:**

Food materials were obtained from different sources, cerelac was made locally, fresh filamentous algae was taken from water bodies, dry filamentous algae was ground to powder after it was dried under shade, and maize pollen was collected from the flowering maize. Each food source type was used to feed three densities of mosquito larvae 20, 60, and 100 in six replicates each. Larval age structure was monitored daily until pupation and subsequently adult emergence. Tetramin was used and taken as a standard food source for *An. gambiae* s.s. larvae feeding in Insectary.

**Results:**

Larval survivorship using maize pollen and Tetramin fish food was statistically insignificant (P = 0.564). However when compared to other food regime survivorship was significantly different with Tetramin fish food performing better than cerelac (*P*<0.001), dry algae (*P*<0.001) and fresh algae (*P*<0.001). The pupation rates and sex ratio of emerging adults had significant differences among the food regimes.

**Conclusion:**

The findings of this study have shown that maize pollen had closely similar nutritional value for larval survivorship to tetramin fish food, a standard larvae food in insectary. Further studies are required to assess the effect of food sources on various life traits of the emerged adults.

## Introduction

*Anopheles gambiae* s.s is medically the most important member of *An.gambiae* complex sibling species due to their anthropophilic behaviour, occurrence of habitats near human dwellings and malaria parasite transmission efficiency [1]–[5]. *An. gambiae* s.s.(hereafter is referred as *An.gambiae*) is a vector of major vector of malaria in sub Saharan Africa [6]. *An. gambiae* breeds in clean shallow water exposed to sunlit with debris [3], [7], [8]. In the recent past, more efforts in elimination and eradication of malaria vectors have been invested in larval source management [9]–[11]. Some of the efforts have been directed at food source type and requirements for the development and growth of *An. gambiae* in order to generate adequate knowledge on natural larval habitats resources for larval developments [12], [13].

The amount and quality of larval food is important for the development, survivorship [14], [15] and adult emergence rate [16]. Larvae of *An. gambiae* feed on the micro-layer of the habitat water surface which have suspended organic particles such as protozoans, and bacteria [17]. In farmland associated habitats, maize pollen were found to have great correlation with larvae abundance [3], [18]–[21]. In natural habitats, algae and bacteria have been found to be the major midgut content for *An. gambiae* larvae [22], and are important food sources [8], [12].

Food availability influences larval development [12], [16], [23], [24] adult emergence, fecundity and survival [14], [16], nutritional reserves [16] and body size [14]. More recently, there has been focus on laboratory larval survivorship and development [14], [16]. The basic larval biology and ecology of *An. gambiae* are considerably undertaken to understand better the larval development on different nutritional opportunities [14], [16]. Some studies have investigated association between food quantity, larval development, larval survivorship and adult emergency rate [14], [25]–[27]. Laboratory studies have shown that larval survivorship is accelerated with abundant food supply [16]. In the field situation, similar trend was observed by Gilbreath and others in western Kenya highlands [12] while in microcosms without food supplement development was delayed with decreased survivorship [12]. Previous studies have demonstrated that larval food quality significantly influences survivorship and adult body size [14], [27], which greatly influences vectorial capacity [9], [14].

Understanding of factors influencing mosquito larval survivorship, adult size and vectorial capacity is critical in designing effective malaria control tools [26]. Information on larval source partitioning [12] and adult longevity [16] can add value to malaria control efforts [3], [4]. Understanding of larvae food source type quality and quantity for *An. gambiae* may be of significant value in rearing mosquitoes in Insectary with high adult productivity using nutritious diet available in local areas.

The objective of this study was to assess the role of five different food regimes on *An. gambiae* larval survivorship, pupation rate, and sex ratio of emerged adult in three different densities of larvae.

## Materials and Methods

### Ethics statement

This study was granted approval by Tropical Pesticides Research Institute (TPRI) research ethical committees as the daily routine activity for routine laboratory mosquito colony maintenance. Maize pollen was collected from farms where farmers had given written consent before collection of Pollen. Participation in the study was voluntary and no any endangered species was involved in this study.

### Preparation of food regimes

Five food sources were prepared for the experiments. These were:

#### Maize pollen

**Figure 1:**
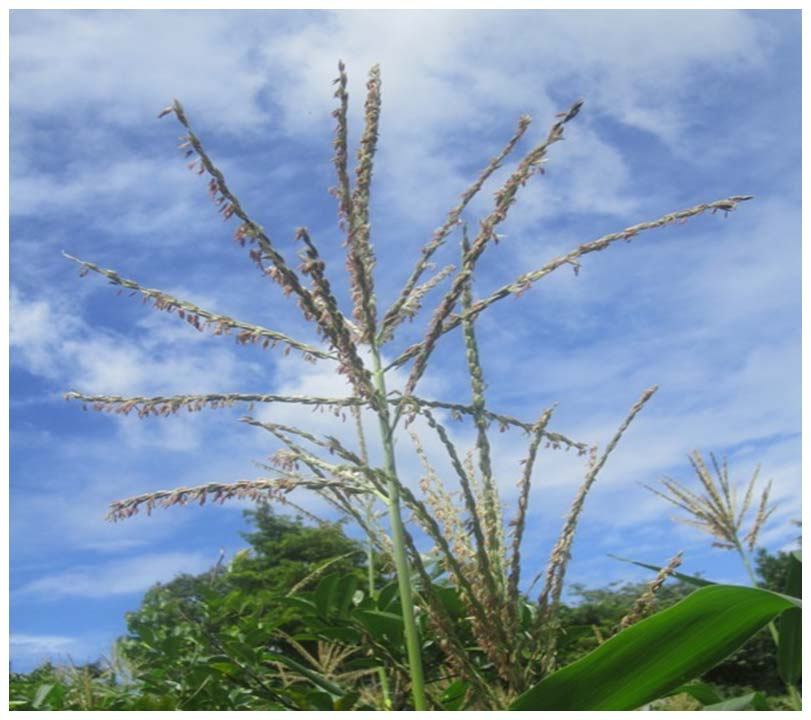
Flowering maize ready for pollen collection.

Maize pollen was collected from the fields with freshly flowering maize (Figure 1) in Ngaramtoni area, near Tropical Pesticides Research Institute and shaken on clean white piece of cloth. Collection was done during maize flowering season (May to June, 2013). The pollen collected was stored in a tight bottle in dry a place ready immediate use. This was done every time when fresh maize pollen was collected. The maize pollen was not stored for more than five days since the collection date. The pollen was fed to mosquito larvae at a rate of 0.003 gm per larva.

#### Filamentous algae

Filamentous algae was collected from drainage ditches in Ngaramtoni area, near Tropical Pesticides Research Institute after rain season (May to June, 2013) and stabilized in basin in laboratory. After stabilization some algae were separated in rearing bowls. When algae in water in laboratory started to develop, water was taken for experiment so larvae can be introduced in a habitat with growing algae which mimics natural habitats. Chlorophyll were determined with a portable turbidimeter (AquaFluor, Sunnyvale, CA, USA). Larvae were introduced only when the chlorophyll content was between 100 and 200 mg/l.

#### Dry filamentous algae powder

Filamentous algae was sampled from drainage ditch in Ngaramtoni area, near Tropical Pesticides Research Institute after rain season (May to June, 2013) and dried under tree shadow. Sampling was done from the same drainage ditch throughout. After thoroughly drying, it was grinded using common pestle. The powder was stored in a tight dry bottle at a room temperature for only five days. The filamentous algae was fed to mosquito larvae at a rate of 0.003 gm per mosquito larvar.

#### Cerelac

Sardines were well dried on the sun burn for three days. Cerelac used for infants was purchased and mixed with ground sardines. Cerelac powder and finely ground sardine powder were mixed in a ratio of 4 part of cerelac and 1 part of sardine powder. The Cerelac was fed to mosquito larvae at a rate of 0.003 gm per mosquito larva. The mixed food was used only for five days. This was made locally following the instruction provided for preparation of cerelac for babies.

#### Tetramin fish food

Tetramin fish food (Tetramin) was purchased from local shop (Tetramin Tropical Flakes-Spectrum Brands, Inc). This is the standard food source used for rearing *An. gambiae* larvae in Insectary [28]. The tetramin fish food was fed to mosquito larvae at a rate of 0.003 gm per mosquito larva. Tetramin was taken as a standard food source for *An. gambiae* s.s. larvae feeding in Insectary.

### Mosquito Rearing

**Figure 2:**
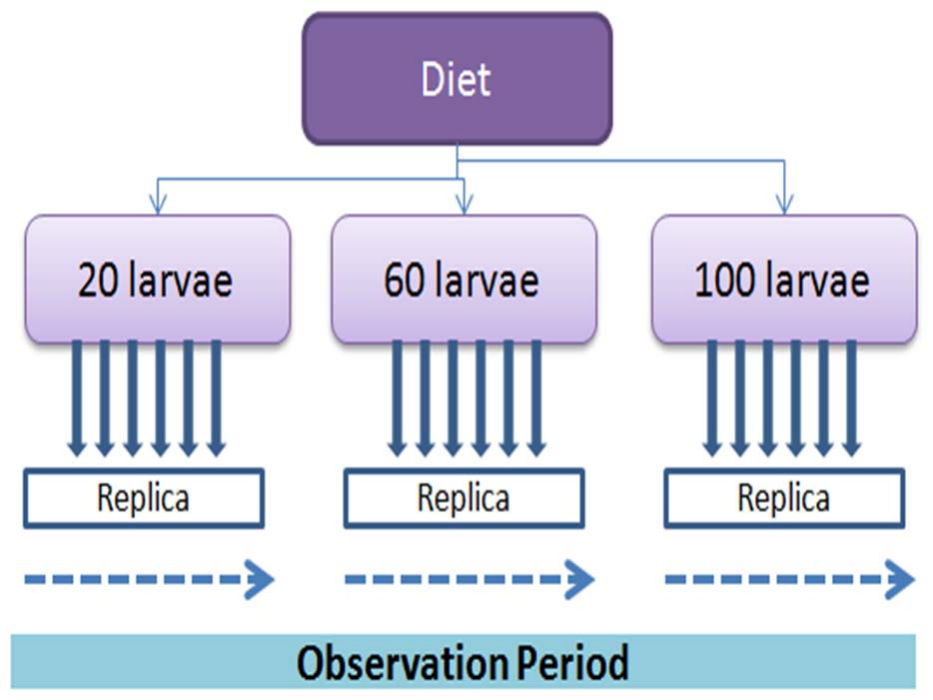
Design of the mosquito rearing experiment with a specific diet.

*An. gambiae sensu stricto* from a colony at the Tropical Pesticides Research Institute (TPRI), Tanzania, was used in this study. This colony was established from a colony sampled in Kisumu Kenya in 1992. First instar *An. gambiae s.s.* larvae were obtained from colony cages and assigned randomly to density treatments of 20, 60 and 100 larvae per rearing tray (5×14×14 cm). Each density was replicated in six times (Figure 2). Each bowl was filled with 0.5 L of water and supplied with fish food (Tetramin), maize pollen, dry algae, cerelac and fresh algae. In each bowl 0.003 gm of Tetramin or Dry algae or maize pollen and cerelac was added in each larvae density and survivorship and larvae instar age structure were recorded daily. Bowls were inspected visually twice a day for the presence of pupae, then collected, counted and held in paper cups with water from the same bowls for emergence. The pupae were separated by according to the respective densities. Batches of males and females from all the three larval densities in different food regimes were counted and recorded in appropriate food regime and density. Larvae and adults were cultured in a climate controlled room at 27±2°C, 70–80% RH, and a photoperiod of 12∶12 (L: D) hrs.

### Data analysis

Performance of diets was assessed by comparing survival rate in each diet to that of Tetramin Fish using log rank test. Effect of larvae density and number of survival days in each cluster and diet were also assessed. Spearman correlation coefficients were estimated for a pair of standard diet and other diets. Cuzick's test of trend was used to assess trend in correlation coefficient as the larvae density increases. Number of days to lose (grow to adult stage) a specific proportion of larvae in a cluster were assessed by indicating time taken to lose 50%, 25% and 100%. Using bar plots these are indicated with median, lower and highest mark. The diet with the shortest bar indicates best performance. Bars for each diet were compared to that of Tetramin Fish.

To conduct survival analysis data were converted to count-time data. Kaplan-Meier curves were used to compare diet-specific survival pattern for each cluster, i.e. indicating all diets and for each pair of diets. Curve for Tetramin Fish was associated with those of other diets. Log-rank significance testing was used to test equality of survivor functions across larva diets in a pairwise with Tetramin Fish and overall. Testing was done for each cluster. All analysis were done in STATA Software (StataCorp. 2007). Significance was considered at 5% level of confidence.

## Results

### Sex ratio

The sex ratio of the emerged adults was calculated for each food type used (Table 1). Only cerelac at density of 20 larvae and maize pollen at a density of 60 larvae had more males than females among emerging as adults. The rest of the experimental density in each food sources had more females than males. However; most of the differences were not statistically significant (P≥0.05).

**Table:** **Table 1.** Sex ratio of emerging of *An. gambiae* s.s. in different densities and food regimes (The number in brackets are the total number of mosquitoes emerged in each density per food regime).

Larval density	Cerelac	Dry algae	Fresh Algae	Maize Pollen	Tetramin
20	45.7 (105)*	57.1 (42)	50 (8)	51.3 (117)	51.4 (111)
60	50.6 (267)	50 (30)	50 (18)	48.9 (284)*	55.3 (329)
100	53.9 (480)	60 (100)	55.6 (18)	52.1 (413)	53.8 (530)

**Note**: The asterisk symbol (*) shows the densities which had more male emerged than females.

### Pupation rate

Overall pupation rate was highest in cerelac followed by maize pollen and tetramin. Both dry algae and fresh algae had the lowest pupation rates (Table 2). The pupation rate in each food type was found to be inversely proportional to larvae density.

**Table:** **Table 2.** Pupation rates of *An. gambiae* s.s. in different densities and food regimes.

Larval density	Cerelac	Dry algae	Fresh Algae	Maize pollen	Tetramin
20	98.3^a^	46.8^b^	45.3^b^	97.2^a^	97.5^a^
60	96.9^a^	41.4^b^	36.7^b^	96.4^a^	91.8^a^
100	95.5^a^	37.6^b^	31.7^b^	94.1^a^	86.75^c^

**Note**: The numbers with the same superscript letters in the same raw they do not differ significantly.

### Survival rates at different densities

**Figure 3:**
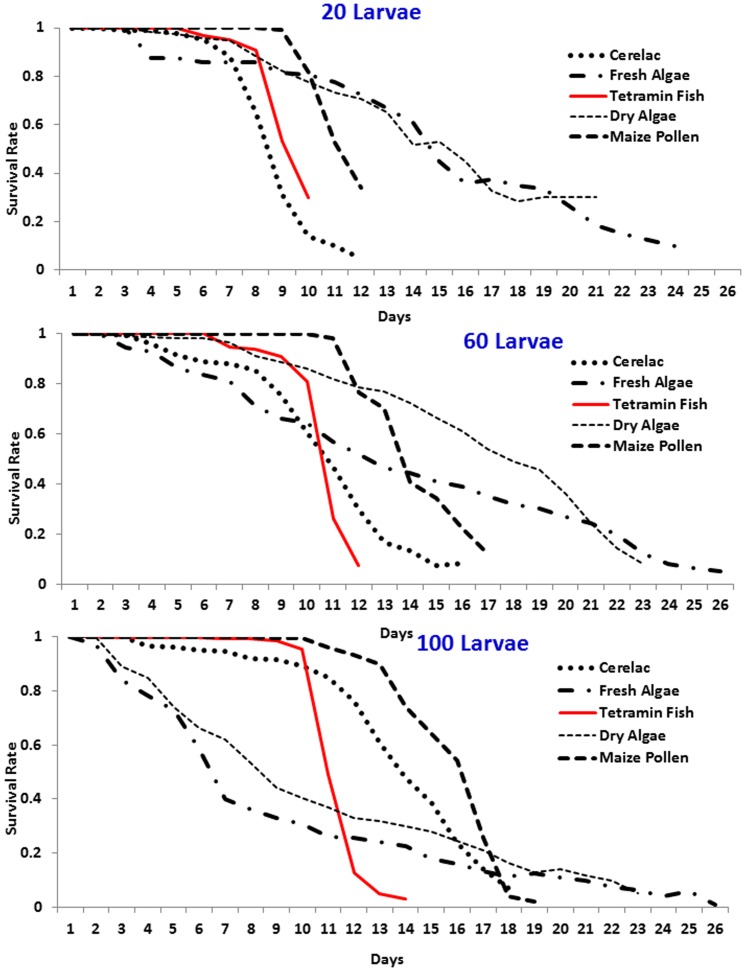
*Anopheles gambiae* s.s. larvae survivorship in different densities and food regimes.

Larval survival rates were found to be larval density and food source dependent. In larvae density of 20, 60 and 100 cerelac, Tetramin fish and maize pollen, constantly had better survivorship than dry algae and fresh algae (Figure 3) (Logrank test, p-value <0.001). Poor survival rate was observed in dry and fresh algae in all density (Figure 3).

**Figure 4:**
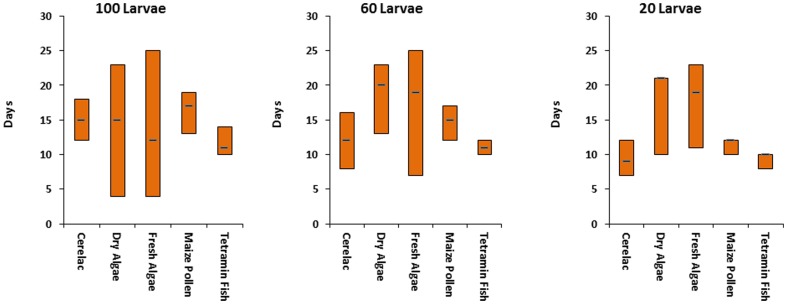
*Anopheles gambiae* s.s. larvae development days to pupae in different densities and food regimes.

Overall larval survivorship was compared by pulling together all densities in each food regimen we observed similar trend in which Tetramin fish, maize pollen and cerelac had better survivorship than dry and fresh algae. The developmental period of the larvae in different diets was significantly influenced by food regime in different densities (Figure 4) with Tetramin fish food showing the shortest time in all density followed by maize pollen. The same trend was observed by comparing the survivorship of all food regimes with standard tetramin fish food (Figure S1, S2 and S3). Survivorship of the larvae instars was found to be food regime and density dependent (Figure S4, Figure S5 and Figure S6). The survivorship correlations between standard food regimes (Tetramin) and other food regimes (Figure S7) showed that the survival rates of Cerelac were highly correlated with that of Tetramin fish food (R^2^ = 0.95) followed by Maize pollen (R^2^ = 0.87) in all density used in these experiments. Dry and fresh algae had lowest correlation, R^2^ = 0.83 and 0.84 respectively.

## Discussion

The findings of this study have demonstrated that, different food regimes have varying impacts on aquatic life stages. Food source influenced factors such as survivorship, developmental time pupation rates and sex ratio. Overall maize pollen and tetramin fish food (the standard laboratory larvae food) had highest positive impact on all factors in aquatic stages of mosquitoes than fresh algae and cerelac, while dry algae powder was least. In previous field studies, maize pollen was showed correlation with larval abundance and high during maize flowering [3], [18]–[21]. In natural larval habitats, algae biomass was found to be the factor for larvae abundance, development and survivorship [3], [12]–[14].

Mosquito larval diet contains several contents, including fatty acid, sugar, amino acids [29], [30]. Other substances such as sterols and nucleotides have been found to be important for larval development, survival and subsequent adult flight efficiency [16], [31]. In natural conditions, larvae have been found to feed on algae, bacteria, phytoplankton and debris [3], [8], [13], [14]. Food quality and quantity availability at aquatic life stages of *An.gambiae* s.l. play important role for the emerging adults life history traits such as fitness, parasite transmission ability and mating [16]. In this study the key findings have shown that, pollen grains taken from flowering maize in the field had higher performance similar to the standard larval food, Tetramin fish food and cerelac which are commonly used to feed mosquito larvae under insectary conditions. In field studies in Ethiopia and Kenya, *An. gambiae* larval and adult densities were found to increase during maize flowering season [3], [18]–[20]. In case of maize pollen results, this confirms that maize pollen have a food component which is critically important like that provided by cerelac and Tetramin fish food. The fresh green filamentous algae abundance in natural habitats has shown to be a main larval food source. In other field studies found algal biomass to be the major content in the larvae guts [13]. This shows that in natural habitat algae have major role in larval survivorship and development. In our experiments in all densities in both fresh and dry algae was found to have low survivorship and pupation rates. This might have been attributed by the algae species used.

We observed that larval life trait parameters, the survivorship and pupation rates of the *An. gambiae* larvae was higher in tetramin fish, cerelac and maize pollen which had no statistical significant difference. this indicates that, the quality of food provided by maize pollen during larval stages supported larvae survivorship even in higher densities [32]. The other larvae development monitoring studies at different larvae densities revealed that, the larvae development is inversely proportional to the larvae developmental duration [9], [32]. In current study, the larval development at density of 100 was well supported by maize pollen at the same rate with tetramin fish food and cerelac. In spite of a known larval density effect on the survivorship and pupation rates, the maize pollen regime had comparatively the same survivorship and pupation rates as cerelac and Tetramin fish food regimes [14]. The survivorship and pupation rates in algae food regime was poorest among all food regimes assessed.

In further observations, the sex ratio of all densities diet were 1∶1 except in density of 20 for cerelac and 60 for maize pollen which was 1∶2 in female to male ratio. In previous studies, the female to male ratio was affected by having more females than males when larvae were crowded and deprived of food [14], [32]. In this study algae food regimes had lowest larvae survivorship but the sex ratio of emerged adults remained similar to other food regimes. The shortcoming of having fewer or same number of males to females might affect the mating success. In natural conditions the ratio of female to male is 1∶3 in *An.gambiae* s.l population [5]. In previous controlled experiments, studies in microcosms have shown the shift in sex ratio when *An. gambiae* larvae occurs in higher density with more females than males [14].

In this study the pupation rate was finding to be inversely proportional to larvae density dependant regardless of the food regimes. Similar results have been reported from other studies conducted in different areas [9], [14], [32]. In habitats the food regime quality determines the number of emerging adult and their body size which determines fecundity, survivorship and host seeking and parasite transmission efficiency [18]–[20], [33]. Mosquito larvae in habitats with high larval density larvae face higher inter and intraspecific competition for food resources and space which in turn reduces larval survival rates and subsequently affects adult life history traits [14], [15], [18], [20], [25]. High larval densities of larvae expose them to waste toxic, crowding chemical cures and physical from other larvae [34], [35]. Understanding larval food regimes impact on adult life history traits in insectary is of importance in designing an effective method for mass rearing of laboratory colony of malaria vector in low cost. Fitness and vigour of the colony should be cross checked often so as to have healthy mosquitoes for different behavioural studies when using different diet regimes in rearing.

## Conclusion

The findings of this study suggest that, the food regime in insectary have an impact on survivorship, pupation and sex ratio of emerging adults and subsequently the vigour and fitness. Food regime nutritional quality, availability and cost are of most important in making successful colony maintenance in insectary.

## Supporting Information

Figure S1 **Survivorship of** ***An.gambiae*** **s.s. larvae in different food regimes compared to tetramin (standard) at density of 20 larvae.**
(TIF)

Figure S2 **Survivorship of** ***An.gambiae*** **s.s. larvae in different food regimes compared to tetramin (standard) at density of 60 larvae.**
(TIF)

Figure S3 **Survivorship of** ***An.gambiae*** **s.s. larvae in different food regimes compared to tetramin (standard) at density of 100 larvae.**
(TIF)

Figure S4 **Growth patterns of larvae in density of 20 in different food regimes.**
(TIF)

Figure S5 **Growth patterns of larvae in density of 60 in different food regimes.**
(TIF)

Figure S6 **Growth patterns of larvae in density of 100 in different food regimes.**
(TIF)

Figure S7 **Correlation between survival rates of larva fed with tetramin and other food regimes by cluster size.**
(TIF)
