# Supplementary figures and images for: Performance of Five Food Regimes on Anopheles gambiae Senso Stricto Larval Rearing to Adult Emergence in Insectary
Source: PLoS One. 2014 Oct 23;9(10):e110671. doi: 10.1371/journal.pone.0110671 (PMC4207715; doi:10.1371/journal.pone.0110671)

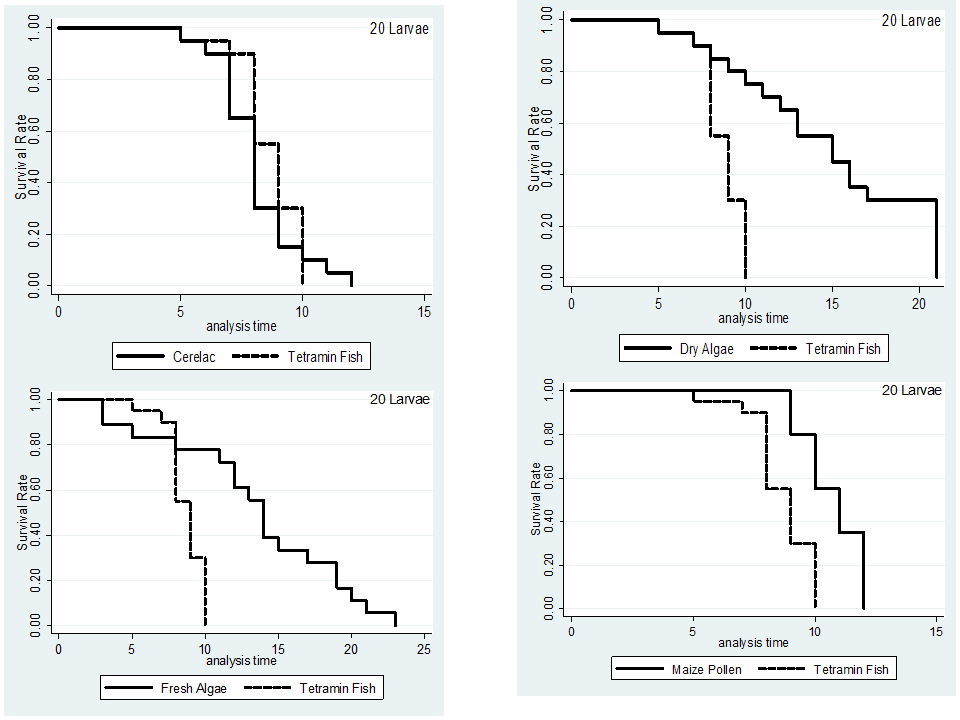

Supplement: Figure S1 — Survivorship of An.gambiae s.s. larvae in different food regimes compared to tetramin (standard) at density of 20 larvae. (TIF) [file pone.0110671.s001.tif]

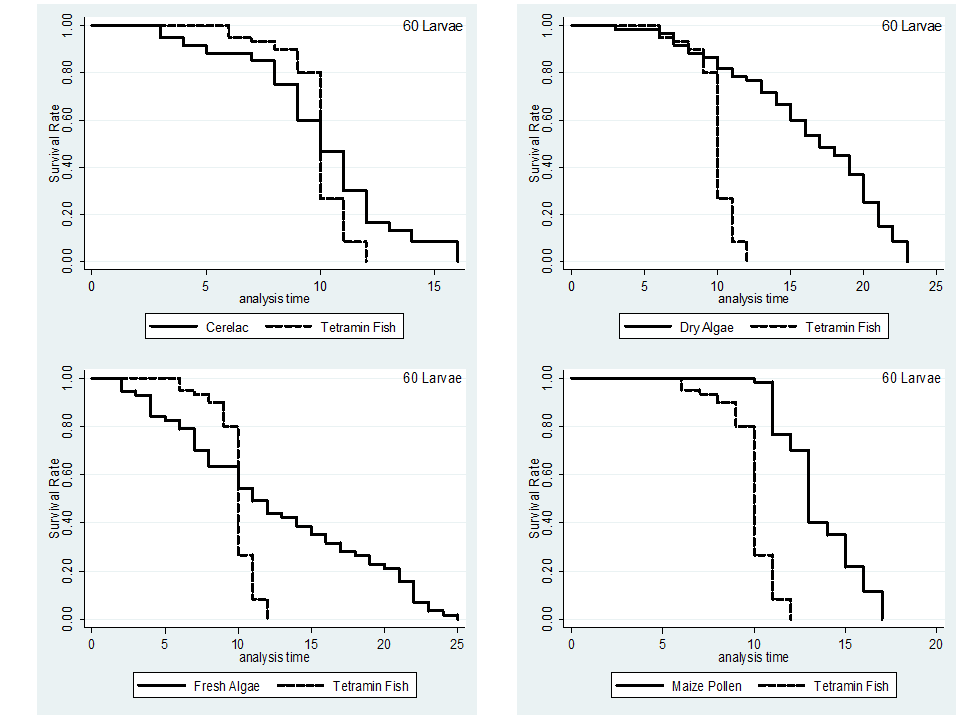

Supplement: Figure S2 — Survivorship of An.gambiae s.s. larvae in different food regimes compared to tetramin (standard) at density of 60 larvae. (TIF) [file pone.0110671.s002.tif]

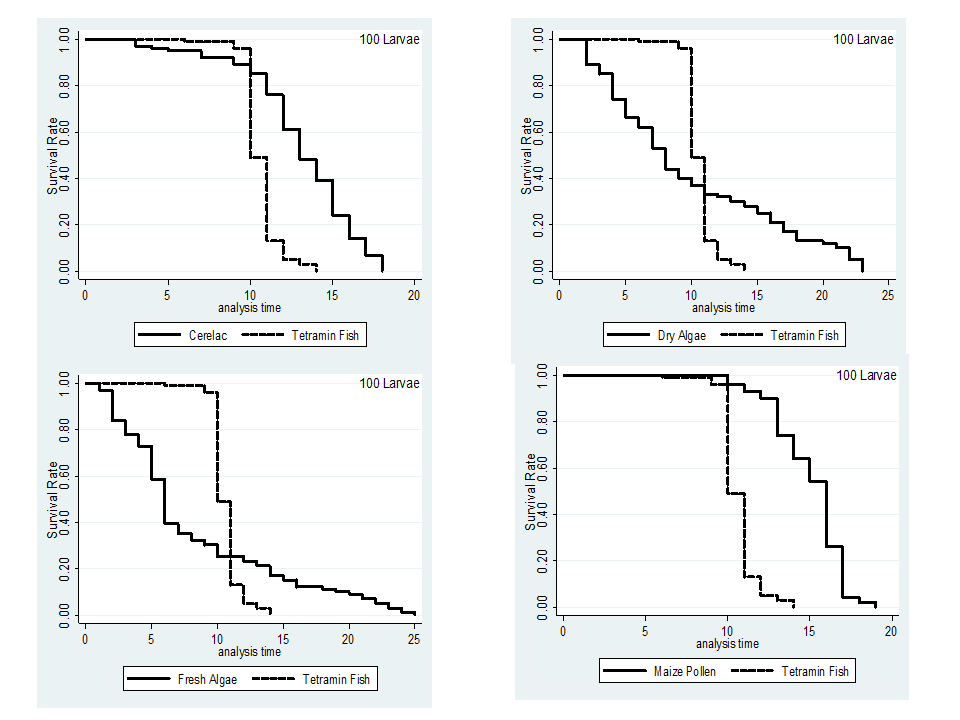

Supplement: Figure S3 — Survivorship of An.gambiae s.s. larvae in different food regimes compared to tetramin (standard) at density of 100 larvae. (TIF) [file pone.0110671.s003.tif]

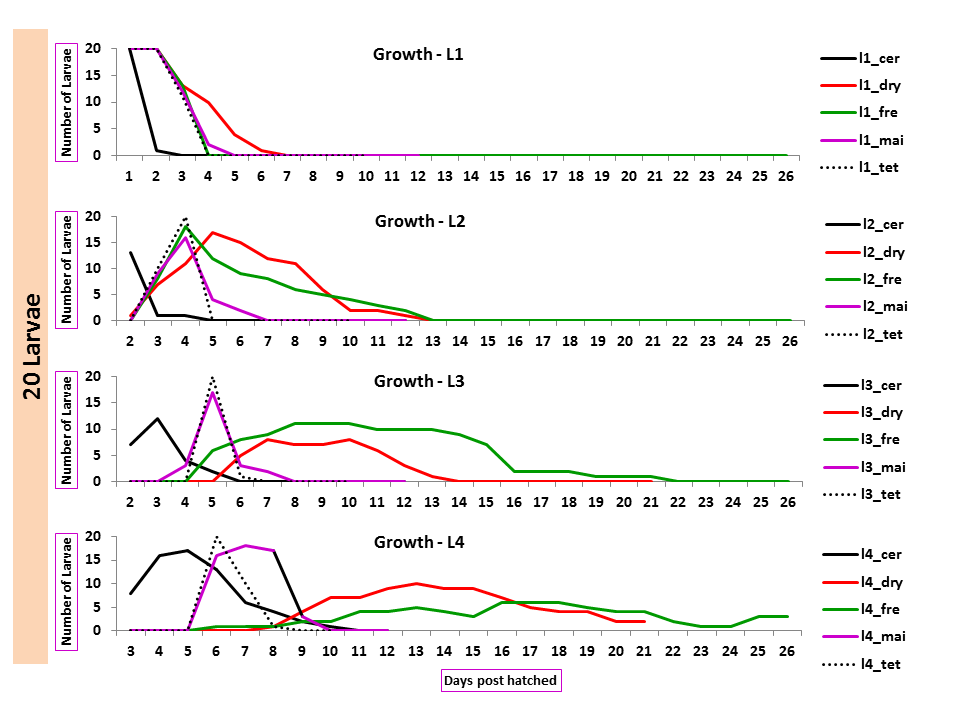

Supplement: Figure S4 — Growth patterns of larvae in density of 20 in different food regimes. (TIF) [file pone.0110671.s004.tif]

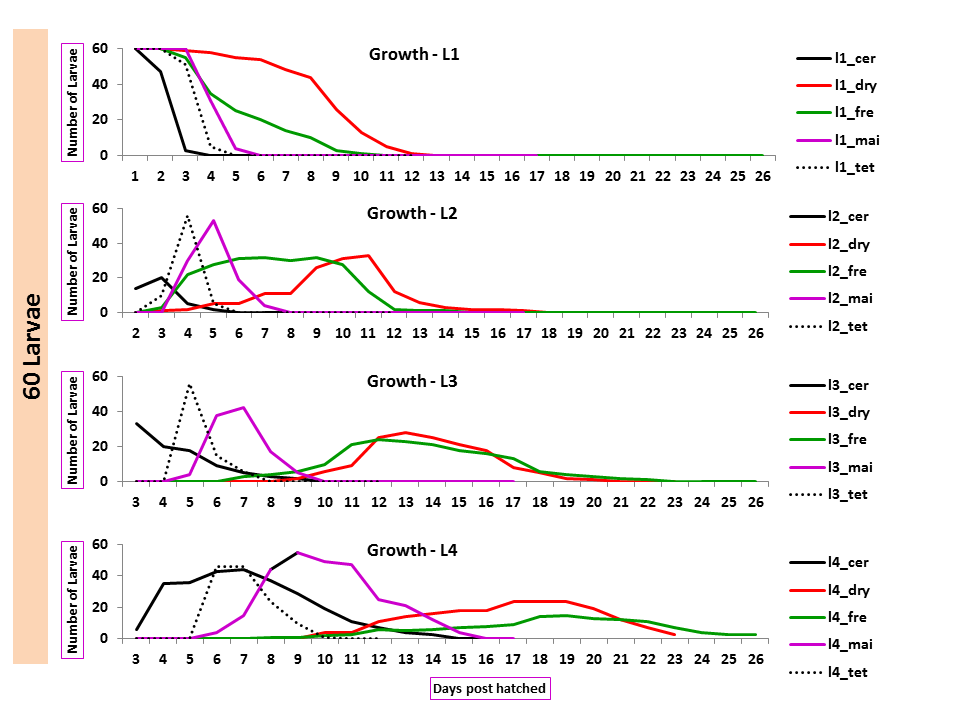

Supplement: Figure S5 — Growth patterns of larvae in density of 60 in different food regimes. (TIF) [file pone.0110671.s005.tif]

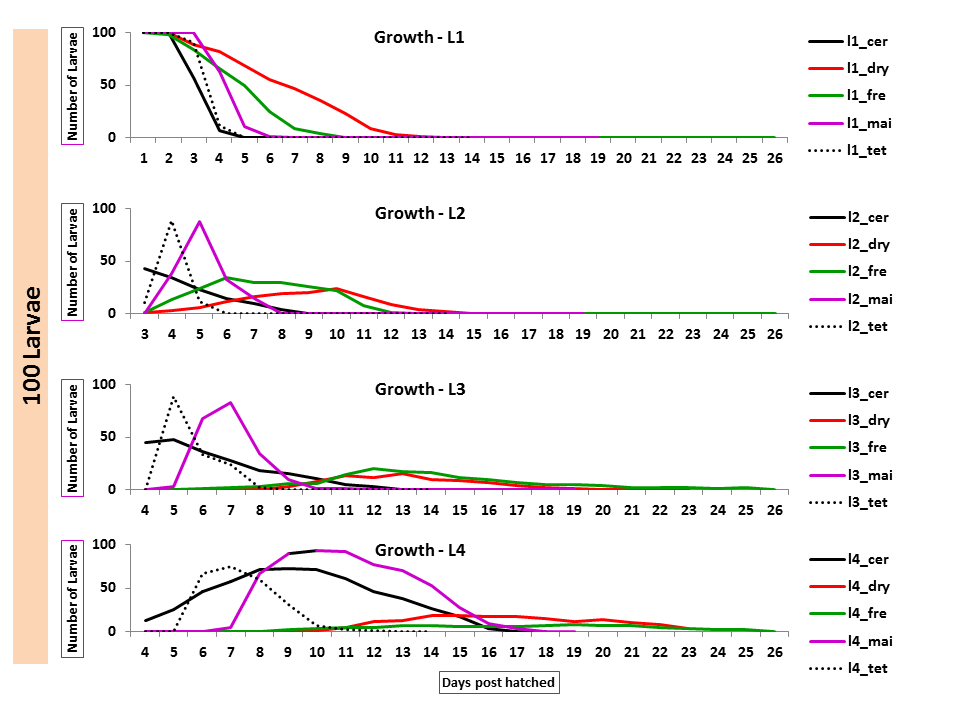

Supplement: Figure S6 — Growth patterns of larvae in density of 100 in different food regimes. (TIF) [file pone.0110671.s006.tif]

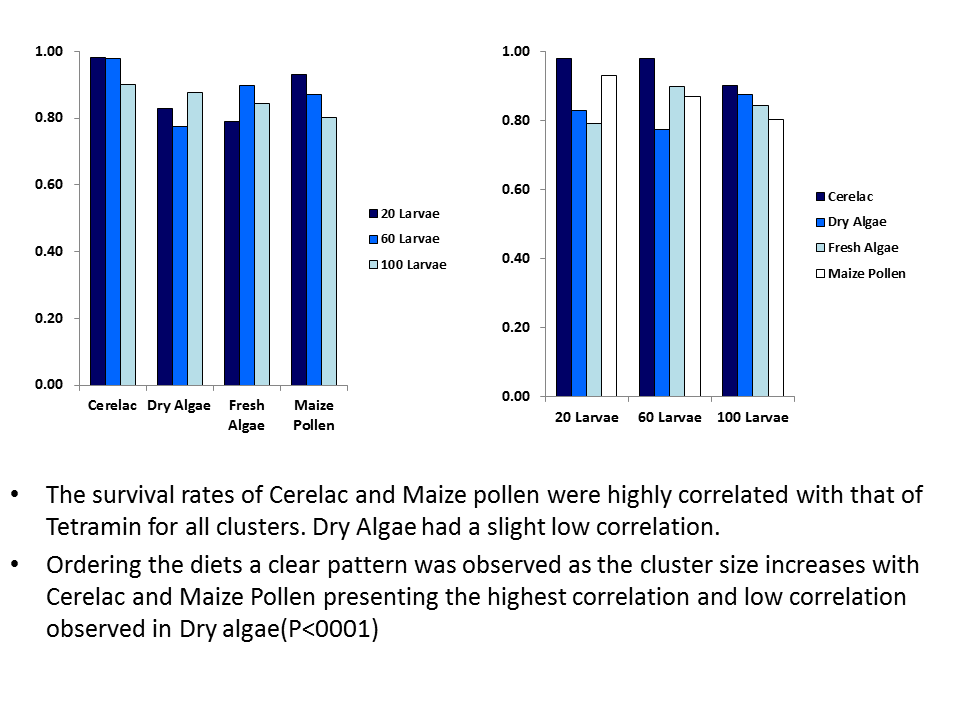

Supplement: Figure S7 — Correlation between survival rates of larva fed with tetramin and other food regimes by cluster size. (TIF) [file pone.0110671.s007.tif]
